# Tilianin: A Potential Natural Lead Molecule for New Drug Design and Development for the Treatment of Cardiovascular Disorders

**DOI:** 10.3390/molecules27030673

**Published:** 2022-01-20

**Authors:** Farrah Syazana Khattulanuar, Mahendran Sekar, Shivkanya Fuloria, Siew Hua Gan, Nur Najihah Izzati Mat Rani, Subban Ravi, Kumarappan Chidambaram, M. Yasmin Begum, Abul Kalam Azad, Srikanth Jeyabalan, Arulmozhi Dhiravidamani, Lakshmi Thangavelu, Pei Teng Lum, Vetriselvan Subramaniyan, Yuan Seng Wu, Kathiresan V. Sathasivam, Neeraj Kumar Fuloria

**Affiliations:** 1 Department of Pharmaceutical Chemistry, Faculty of Pharmacy and Health Sciences, Royal College of Medicine Perak, Universiti Kuala Lumpur, Ipoh 30450, Perak, Malaysia; farrahsyzn@gmail.com (F.S.K.); peiteng1013@gmail.com (P.T.L.); 2 Faculty of Pharmacy, AIMST University, Bedong, Kedah 08100, Malaysia; azad@aimst.edu.my; 3 School of Pharmacy, Monash University Malaysia, Bandar Sunway, Selangor 47500, Malaysia; gan.siewhua@monash.edu; 4 Faculty of Pharmacy and Health Sciences, Royal College of Medicine Perak, Universiti Kuala Lumpur, Ipoh 30450, Perak, Malaysia; najihah.izzti@gmail.com; 5 Department of Chemistry, Karpagam Academy of Higher Education, Coimbatore 641021, Tamil Nadu, India; ravisubban@rediffmail.com; 6 Department of Pharmacology, College of Pharmacy, King Khalid University, Abha 62529, Saudi Arabia; kumarappan@kku.edu.sa; 7 Department of Pharmaceutics, College of Pharmacy, King Khalid University, Abha 61421, Saudi Arabia; ybajen@kku.edu.sa; 8 Department of Pharmacology, Sri Ramachandra Faculty of Pharmacy, Sri Ramachandra Institute of Higher Education and Research (DU), Porur, Chennai 600116, Tamil Nadu, India; srikanth.j@sriramachandra.edu.in (S.J.); arulmozhidhiravidamani55@gmail.com (A.D.); 9 Center for Transdisciplinary Research, Department of Pharmacology, Saveetha Dental College and Hospital, Saveetha Institute of Medical and Technical Sciences, Saveetha University, Chennai 600077, India; lakshmi@saveetha.com; 10 Faculty of Medicine, Bioscience and Nursing, MAHSA University, Jalan SP 2, Bandar Saujana Putra, Jenjarom 42610, Selangor, Malaysia; drvetriselvan@mahsa.edu.my; 11 Centre for Virus and Vaccine Research, School of Medical and Life Sciences, Sunway University, Subang Jaya 47500, Malaysia; sengwu_21@yahoo.com; 12 Department of Biological Sciences, School of Medical and Life Sciences, Sunway University, Subang Jaya 47500, Malaysia; 13 Faculty of Applied Sciences, AIMST University, Bedong, Kedah 08100, Malaysia; skathir@aimst.edu.my

**Keywords:** tilianin, cardiovascular disorders, cardioprotection, molecular mechanism, drug-likeness, drug development

## Abstract

Cardiovascular disorders (CVDs) are the leading risk factor for death worldwide, and research into the processes and treatment regimens has received a lot of attention. Tilianin is a flavonoid glycoside that can be found in a wide range of medicinal plants and is most commonly obtained from *Dracocephalum moldavica*. Due to its extensive range of biological actions, it has become a well-known molecule in recent years. In particular, numerous studies have shown that tilianin has cardioprotective properties against CVDs. Hence, this review summarises tilianin’s preclinical research in CVDs, as well as its mechanism of action and opportunities in future drug development. The physicochemical and drug-likeness properties, as well as the toxicity profile, were also highlighted. Tilianin can be a natural lead molecule in the therapy of CVDs such as coronary heart disease, angina pectoris, hypertension, and myocardial ischemia, according to scientific evidence. Free radical scavenging, inflammation control, mitochondrial function regulation, and related signalling pathways are all thought to play a role in tilianin’s cardioprotective actions. Finally, we discuss tilianin-derived compounds, as well as the limitations and opportunities of using tilianin as a lead molecule in drug development for CVDs. Overall, the scientific evidence presented in this review supports that tilianin and its derivatives could be used as a lead molecule in CVD drug development initiatives.

## 1. Introduction

The circulatory system, also known as the cardiovascular system, is the organ system that distributes blood to and from all regions of the body through vessels. It is the most important component of the human body consisting of the heart, blood vessels, veins, and arteries circulating throughout the human body. The circulatory system is operated by the heart which is responsible for distributing nutrients, oxygen, hormones, and waste products from cells throughout the body. A resting heart circulates approximately five litres of blood every minute throughout the body. The common cause of death related to the cardiovascular system worldwide is known as cardiovascular disease (CVD). CVD is a broad term that encompasses a variety of various conditions. Several of them may occur concurrently or may even result in the development of additional disorders or diseases. According to the World Health Organization (WHO), globally, CVD constitutes a significant cause of death, claiming 17.9 million lives in 2019, and accounting for 32% of all premature deaths documented. Heart attacks and strokes were responsible for 85% of these deaths [1]. By 2030, the WHO estimates that CVD-related deaths may increase to 23.6 million/year by 2030. CVD is known to affect both men and women equally [1].

There are numerous complications that can occur within the cardiovascular system, including endocarditis, rheumatic heart disease and abnormalities in the conduction system. CVD refers to four entities, which are: (1) coronary artery disease (CAD), commonly known as coronary heart disease (CHD), is a condition caused by reduced myocardial perfusion, which leads to angina, myocardial infarction (MI) and/or heart failure; (2) cerebrovascular disease is a broad term that encompasses stroke and transient ischemic attack (TIA); (3) peripheral arterial disease (PAD) is a condition that affects the peripheral arteries, particularly arterial illness affecting the limbs, which can cause claudication and (4) aortic atherosclerosis that includes thoracic and abdominal aneurysms [2]. Cardioprotection (CP) is a strategy for protecting the heart against various insults such as ischemia, ischemia/reperfusion injury (I/R) and chemical, metabolic and physical stresses, as well as lowering the risk of heart failure (HF) and death. Cardioprotection is defined as “all systems and techniques that contribute to the heart’s preservation by minimising or even preventing myocardial damage” [3]. Since acute ischemia-reperfusion injury causes myocardial damage, cardioprotection is an endogenous system that can decrease or prevent this damage, acting by reducing myocardial damage caused by coronary artery surgery and acute MI, both of which have high morbidity and cause death. In addition to current best-practice treatment, new cardioprotective treatments must be able to prevent or reduce myocardial damage. Cardioprotection in ischemic heart disease has come a long way in the last 40 years where re-oxygenating a blocked artery can minimise the severity of a MI.

**Figure 1:**
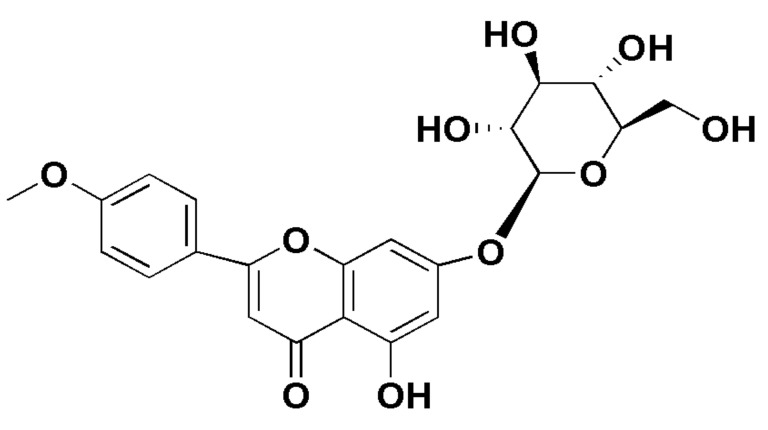
Chemical structure of tilianin.

Medicines from natural products or herbs are becoming increasingly common in the medical field in the treatment of diseases defined as phytomedicine. Since ancient times, phytomedicines show a wide range of biological actions and is therefore utilised in the prevention and treatment of diseases globally [4]. The development of a novel drug molecule is a time-consuming and expensive process. One of the most important tasks in the drug discovery and development process is identification of a lead molecule, i.e., a molecule with a particular degree of potency that can be chemically changed to improve its biological activity, alter the metabolism, and pharmacokinetic profiles. Natural products from terrestrial sources (plants and fungus), microorganisms, and marine species, have a wide range of such molecules with structural diversity, making them a good source of new drug leads and therapeutic agents [5,6,7,8,9]. Researchers are intrigued by the prospect of finding novel biologically active compounds in natural products, particularly in herbs, for the treatment of diseases, especially CVD. Tilianin (acacetin-7-glucoside) is an active flavonoid glycoside (Figure 1) obtained from numerous medicinal plants and especially from *Dracocephalum moldavica*. It is also found in various common medicinal herbal plants including *Agastache Mexicana, Agastache rugosa*, *Dracocephalum moldavia*, *Dracocephalum tanguticum*, *Dracocephalum moldavica*, *Lygodium japonicum* and *Discocleidion rufescens* [10,11]. These plants are found in the East Asia region including China, Japan, Korea and Mexico. It was reported for wide range of biological activities including antidiabetic [12], anti-inflammatory [13], antioxidant [14], anti-depressant [15], cardioprotection [16,17] and neuroprotection [18].

**Figure 2:**
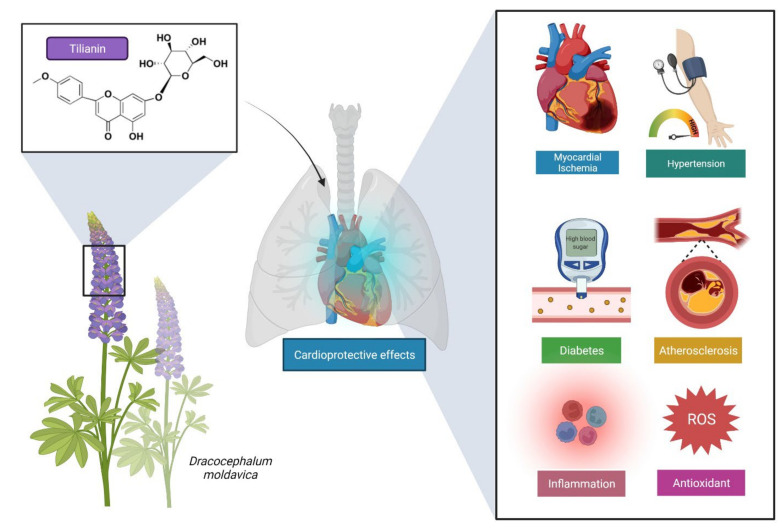
Tilianin, obtained mainly from *Dracocephalum moldavica* acts as a cardioprotectant and lowers the risk of CVDs.

Scientific evidence clearly indicated that tilianin is a potential natural compound against CVDs such as atherosclerosis, coronary heart disease, angina pectoris, hypertension and myocardial ischemia (Figure 2). Hence, the present review aimed to provide the experimental evidence of tilianin against CVDs. The toxicity profile, as well as the physicochemical and drug-likeness properties, were also highlighted in this review. We also conducted molecular docking studies with selected proteins to validate its cardioprotection mechanism in order to strengthen this review. Along with that, we discuss tilianin-derived molecules, as well as the limitations and possible opportunities of using tilianin as a lead molecule in the drug development for CVDs.

## 2. Cardioprotective Effect of Tilianin

CVDs contribute to a significant cause of death in the world. Based on epidemiological research, many important risk factors for heart diseases are of environmental and biological origins [19]. The major cause of MI is oxidative damage as induced by cholesterol in the oxidation of low-density lipoprotein [20]. Jiang et al. [21] demonstrated that tilianin can ameliorate oxygen–glucose deprivation/re-oxygenation (OGD/R)-induced cardiomyocyte injury and maintain cardiac function in coronary artery ischemia/reperfusion (I/R)-injured hearts. Tilianin interacts with calcium/calmodulin-dependent protein kinase II (CaMKII) in cardiomyocytes injured by myocardial ischemia reperfusion injury (MIRI), regulating the expressions of p-CaMKII and ox-CaMKII with an efficient binding performance and strong binding score as well as inhibiting the expressions p- and ox-CaMKII. Importantly, CaMKII abolished tilianin-mediated recovery of OGD/R-induced cardiomyocyte injury and the maintenance of cardiac function in I/R-injured hearts at the expense of mitochondrial function protection. Furthermore, tilianin’s protective effects on mitochondrion-associated pro- and anti-apoptotic protein balancing as well as c-Jun N-terminal kinase (JNK)/nuclear factor (NF-) *κ*B-related inflammation suppression were both abolished following the pharmacological inhibition of CaMKII [21].

In another study, administration of tilianin to rats reduced MI by: (1) improving the pathological morphology of the myocardium; (2) increasing the contents of ATP and NAD; (3) decreasing ADP and AMP levels as well as the ratio of AMP/ATP; (4) reducing the level of ROS and MDA and (5) up-regulating the expressions of AMPK, SIRT1, PGC-1α [22]. Furthermore, pre-administration of high dose tilianin reduced lactate dehydrogenase (LDH), malondialdehyde (MDA) and creatinine kinase-MB (CK-MB) release while increasing plasma superoxide dismuthase (SOD) levels and considerably reducing infarct size [17]. Moreover, western blot analysis revealed an increase in Bcl-2 and XIAP expressions in the myocardium, as well as a decreasing the expressions of Bax, Smac/Diablo, HtrA2/Omi as well as cleaved caspases-3, -7 and -9. Interestingly, the levels of phosphorylated Akt and PI3K were found to be increased in the presence of a high dose of tilianin.

**Figure 3:**
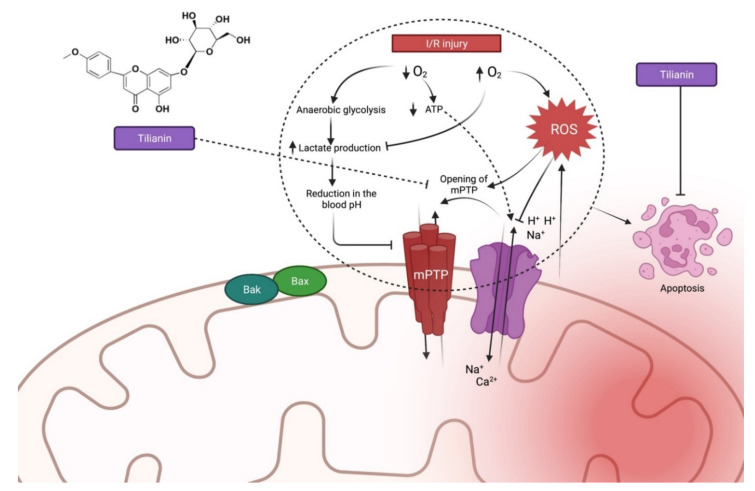
Possible mechanism of action of tilianin during I/R injury [21,22,23,24,25]. In the ischemic phase, less oxygen is produced, resulting in an increase in lactate production, lowering the blood pH. Reduced oxygen also results in the opening of the mPTP, which increases ROS formation during reperfusion. The series of events affects intracellular H^+^, Na^+^ and Ca^2+^ distribution, eventually resulting in apoptosis. Abbreviations: ROS, Reactive oxygen species; mPTP, Mitochondrial permeability transition pore, Bax, Bcl-2 Associated X-protein; Bak, BCL2 Antagonist/killer.

In rats, tilianin pretreatment has been found to inhibit apoptosis following I/R injury [23]. Tilianin also reduces mitochondrial damage, blocked 1-methyl-4-phenyl-1,2,3,6-tetrahydropyridine (mPTP) opening and increased ATP generation. Additionally, the concentration of calcium (Ca^2+^) and ROS in the mitochondria was also reduced. Based on an apoptosis protein analysis by Wang et al. [23], treatment with tilianin resulted in the downregulation of apoptosis-inducing factor (AIF), as well as the suppression of cytochrome c leakage and caspase-3 activation. When tilianin was administered to MIRI rats, it enhanced ATPase activity and decreased serum nitric oxide (NO) and myocardial nitric oxide synthase (NOS) activities, both of which are important in the regulation of endothelial function. In addition, tilianin caused dose-dependent decreases in endothelin-1 and thromboxane B2 levels, as well as increases in calcitonin gene-related peptide and 6-keto prostaglandin F1a levels. Tilianin treatment reduced apoptosis, as confirmed by the increased Bcl 2 expression and decreased Bax and caspase 3 mRNA expression levels [24]. Similarly, pretreatment with tilianin in rats increased the myocardium’s ATP levels and protected the microstructures and functions of mitochondria in a dose-dependent manner. Additionally, tilianin’s cytoprotective action has been verified in vivo and in the H9c2 cardiomyoblast cell line as confirmed by the increased mitochondrial activity, regulation of Ca^2+^ and ROS and decreased caspase-3 and AIF productions in the cytoplasm. The study by Yuan et al. [25] further suggests tilianin’s clinical utility for cardiomyocyte and mitochondrial protection by suppressing myocardium energy metabolism and apoptosis during MIRI. Figure 3 summarises the possible mechanism of action of tilianin during I/R injury based on the abovementioned findings.

**Figure 4:**
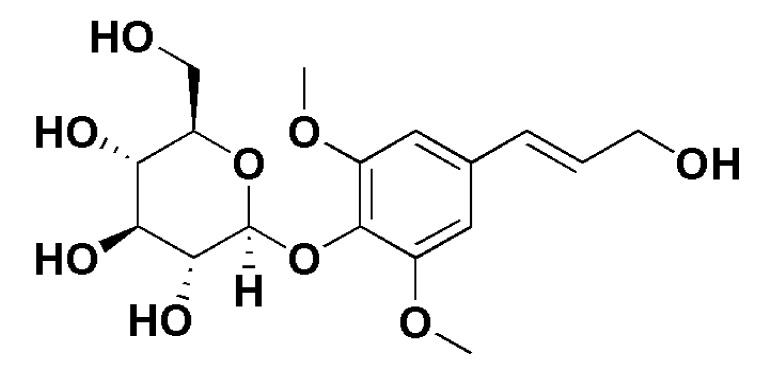
Chemical structure of syringin.

In diabetic rats and hyperglycemic-H9c2 cells, Yao et al. [26] investigated the combined effect of tilianin and syringin (50 and 60 mg/kg, i.p. respectively for eight weeks) on diabetic cardiomyopathy. They evaluated the cardiac function, inflammation, oxidative stress, apoptosis and mitochondrial function, as well as the contribution of TLR4/NF- *κ*B/NLRP3 and PGC. The combination of tilianin and syringin (Figure 4) prevented the diabetic-induced cardiac functional, biochemical and histological alterations in diabetic cardiomyopathy in a synergistic manner. The conferred protection was further strengthened by crosstalk between the TLR4/NF-*κ*B/NLRP3 and PGC1/SIRT3/mitochondrial pathways. All of the above findings suggested that tilianin could help in preventing ischemic heart disease, such as myocardial infarction, by inhibiting inflammatory reactions and oxidative stress [26].

## 3. Atheroprotective Effect of Tilianin

Atherosclerosis is a multiphase process that significantly contributes to CHD and stroke and is the leading cause of mortality globally. Atherosclerosis has several pathophysiological processes and causes, the most significant of which are chronic inflammation and endothelial dysfunction [27]. Consequently, minimising endothelial damage may be a critical therapeutic strategy for atherosclerosis control and therapy. In vitro and in vivo studies conducted by Nam et al. [28] showed that tilianin has anti-atherogenic properties where the mice fed with a high-cholesterol diet supplemented with tilianin had considerably smaller lesions and lower cytokine levels when compared to control, while their serum cholesterol levels remained unchanged. In another study, the levels of TNF-α and IL-1β mRNA levels in primary cultured peritoneal macrophages from *Ldlr*−/*−* mice in response to LPS treatment were ameliorated by co-treatment with tilianin. Furthermore, electrophoretic mobility shift and NF-κB promoter experiments revealed that tilianin suppressed NF-κB activation. Tilianin prevented IκB kinase activation and the subsequent phosphorylation and degradation of IκBα protein upstream of NF-κB activation [28].

**Figure 5:**
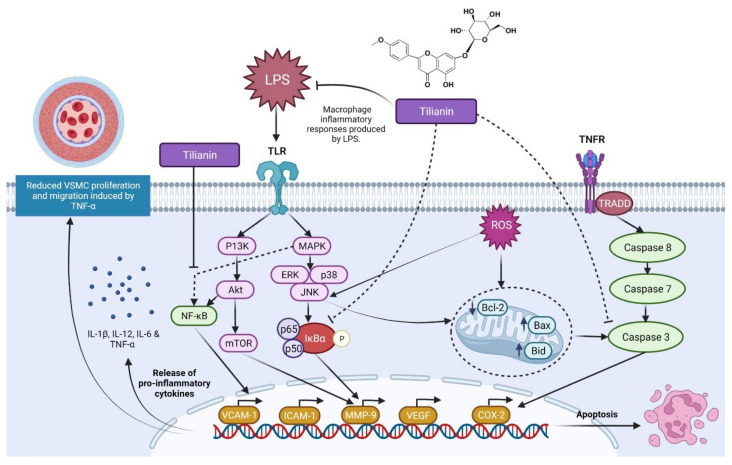
Possible mechanism of action of tilianin against atherosclerosis [29,30]. Tilianin inhibits the activation of NF-κB, as well as the activation, phosphorylation and degradation of IkB kinase. This bioactive compound also inhibits TNF-induced VSMC proliferation and migration while lowering LPS-induced macrophage inflammation. Abbreviations: TLR, Toll-like receptor; TNFR, Tumor necrosis factor receptor; MAPK, Mitogen-activated protein kinase; P13K, Phosphatidylinositol-3-Kinase; ERK, Extracellular signal-regulated kinase; JNK, c-Jun N-terminal kinases; IκBα, Nuclear factor of kappa light polypeptide gene enhancer in B-cells inhibitor alpha; Akt, Ak strain transforming; NF-κB, Nuclear factor kappa-light-chain-enhancer of activated B cells; mTOR, Mammalian target of rapamycin; VCAM-1, Vascular cell adhesion protein 1; ICAM-1, Intercellular Adhesion Molecule 1; MMP-9, Matrix metallopeptidase 9; VEGF, Vascular endothelial growth factor; COX-2, Cyclooxygenase-2; Bcl-2, B-cell lymphoma 2; Bax, Bcl-2 Associated X-protein; Bid, BH3 interacting-domain death agonist; IL-1β, -12, -6, Interleukin-1 beta,-12,-6; TNF-α, Tumour necrosis factor alpha; VSMC, Vascular smooth muscle cell.

Shen et al. [29] investigated the anti-atherosclerotic mechanism of tilianin by developing in vitro models with macrophages, vascular smooth muscle cells (VSMC) and human umbilical vein endothelial cells, all of which contribute to atherosclerosis progression. Tilianin decreased TNF-α levels, generated VSMC proliferation and migration while suppressing LPS-induced inflammatory responses on macrophages. Furthermore, tilianin’s anti-inflammatory action on macrophages and VSMCs was demonstrated to be mostly contributed by the downregulation of the TNF-α/NF-κB pathway. Moreover, their findings showed that tilianin reduced the development of oxidized low-density lipoprotein (ox-LDL)-induced foam cells in macrophages by suppressing SR-A1 mRNA expression and inducing the expression of genes involved in cholesterol efflux, such as SRB-1 and ABCA1. Cao et al. [30] investigated the effects of tilianin on rat vascular smooth muscle cells (VSMCs) induced with angiotensin II on the proliferation, migration and the TGF-β/Smad signalling pathway (Ang II). In VSMCs induced by Ang II, tilianin suppresses the proliferation and expression of intracellular PCNA in a dose-dependent manner. Tilianin also inhibits the migration and expression of intracellular ICAM-1, VCAM-1, MMP-2 and MMP-9 in a dose-dependent manner. Additionally, tilianin suppresses TGF-β, Smad2, Smad3, Smad2/3 and PSmad2/3 in Ang II-induced VSMCs. Overall, tilianin’s inhibitory properties support its usage in the prevention and treatment of atherosclerosis. Based on the aforesaid data, Figure 5 summarises the possible mechanism of action of tilianin against atherosclerosis.

## 4. Antihypertensive and Vasorelaxant Effects of Tilianin

**Figure 6:**
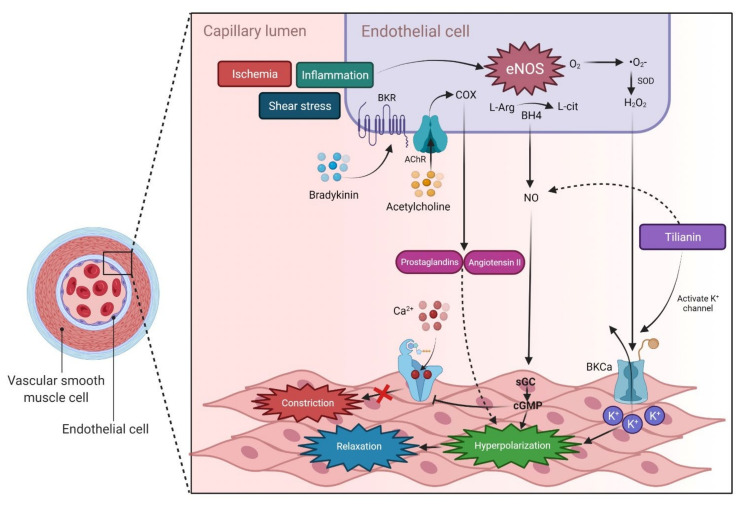
The endothelial cell is involved in a variety of physiological processes, including the regulation of VSMC via a balance of vasodilators and vasoconstrictors. After being activated by endogenous stimuli such as acetylcholine, bradykinin, inflammation, shear stress and ischemia, NO diffuses into smooth muscle cells and sGC to become cGMP, which promotes smooth muscle relaxation. Tilianin stimulates K^+^ channels opening in vascular smooth muscle cells and NO release, resulting in hyperpolarization of the cell membrane and tissue relaxation [34]. Abbreviations: VSMC, Vascular smooth muscle cell; eNOS, Endothelial nitric oxide synthase; COX, Cyclooxygenase; SOD, Superoxide dismutases; O_2_, Oxygen; •O2^−^, superoxide; H_2_O_2_, Hydrogen peroxide; L-Arg, L-arginine; BH4, Tetrahydrobiopterin; L-cit, L-citrulline; AChR, Acetylcholine receptor; BKR, Bradykinin receptor; NO, Nitric oxide; BKCa, Ca^2+^-activated K^+^ channel; sGC, Soluble guanylate cyclase; cGMP, Cyclic guanosine monophosphate; K^+^, Potassium.

Despite recent advancements in the prevention, detection and treatment of high blood pressure, hypertension remains a major public health concern [31]. Hypertension affects approximately one billion individuals worldwide [32]. It is linked with an increased incidence of stroke, coronary heart disease, congestive heart failure and end-stage renal disease. Its occurrence can be decreased by reducing risk factors for its development, which include obesity, physical inactivity, smoking, poor potassium intake and excessive alcohol consumption. Uncontrolled hypertension raises the chance of serious health issues and is a major trigger for MI, blindness, renal disease and stroke [33]. Administration of tilianin (50 mg/kg, single dose, p.o.) resulted in significant reduction in systolic and diastolic blood pressures. Tilianin induces relaxation primarily through an endothelium-dependent pathway, likely due to NO release, as well as an endothelium-independent pathway by activating K^+^ channels, both of which confer the antihypertensive effect [34] (Figure 6).

Furthermore, in aortic rings pre-contracted with noradrenaline (NA, 0.1 µM) and the presence of serotonin (5-HT, 100 µM), tilianin (0.002–933 µM) induced significant relaxation in concentration- and endothelium-dependent and -independent manners. Moreover, tilianin (130 µM) caused a significant shift to the left in the sodium nitroprusside (SNP; 0.32 nM–0.1 µM)-induced relaxation curve. Tilianin also caused significant in vitro NO overproduction in the rat aorta. Pre-treatment with tetraethylammonium (TEA, 5 µM) and 2-aminopyridine (2-AP, 0.1 µM) altered the relaxant curve induced by tilianin to the right [35]. Moreover, the antihypertensive impact of tilianin was dose-dependent with the calculated effective dose 50% (ED_50_) (53.51 mg/kg) being lower than the calculated lethal dose 50% (LD_50_) (6624 mg/kg), implying a wide range of pharmacology–toxicology patterns [10]. The findings indicate that tilianin should be explored further as an antihypertensive agent in randomized clinical trials. Carmona-Castro et al. [35] and Hernández-Abreu et al. [36] reported that the methanolic extract of aerial parts of *Agastache mexicana* has higher tilianin concentrations with potential vasorelaxant effects indicating that various parts of the plants have differing effects. The in vivo studies investigated the efficacy of tilianin against CVDs were summarized in Table 1.

## 5. Overview of the Mechanisms of Action for Tilianin against CVDs

The basic mechanism of tilianin in protecting the heart from CVDs in this review include: (1) vascular protection; (2) blood pressure modulation; (3) cholesterol level reduction; (4) platelet-blocking function; (5) lipid-metabolism regulation; (6) oxidative stress reduction and (7) ischemia or reperfusion reduction.

Pre-treatment with tilianin confers cardioprotective effect, with PI3K/Akt signalling playing a key part in the process as indicated by the improved cardiac damage recovery and a lower rate of myocardial apoptosis. It is critical to avoid apoptosis by limiting the cardiac damage produced by myocardial ischemia reperfusion injury (MIRI). MIRI is a type of tissue damage that develops during early coronary flow and returns to the heart promptly after ischemia, increasing myocardial injury. Based on previous research, tilianin has a cardioprotective effect and its fundamental mechanism is mitochondria-dependent Bax/Bcl-2, cytochrome c, caspase and PI3K/Akt signalling that regulates LDH, MDA, CK-MB and SOD factors. Furthermore, a study by Zeng et al. [17], stated that tilianin recovers MIRI by initiation of PI3K/Akt signalling which prevents myocardial apoptosis (caspase-3). Tilianin has an anti-atherosclerotic effect by repairing tissue as well as controlling inflammation. It is a common risk for an individual with a heart condition or MI, to have an underlying atherosclerotic condition where hypertension, diabetes and dyslipidemia are important risk factors. In addition, atheroprotection is another area where tilianin has potential since tilianin ameliorates all inflammatory responses, oxidative stress, lipid dysregulation and epigenetic disorders that contribute to the condition. Based on Nam et al. [28], the mechanism involved is regulation of inflammatory cytokines (TNF-α, IL-1β) and TGF-β/Smad/NF-κB signallings. In addition, tilianin also confers antihypertensive and vasorelaxant effects [34].

Hypertension is a common disorder that contributes to MI, stroke, kidney debilities and blindness [33]. A study performed on *Agastache mexicana* extract, an important source of crude tilianin, revealed to act as anti-hypertensive and a vasorelaxant [34]. Furthermore, tilianin cause a dose-dependent reduction in systolic and diastolic blood pressures through endothelium-independent mediated by potassium (K^+^) channel opening and NO/cGMP pathways. All the evidence points to tilianin’s potential protective effect against CVD disorders since tilianin can regulate oxidative stress, lipid metabolism, inflammation, exert positive influences on macrophage, arterial endothelial cells and prevent VSMC from excessive proliferation. Nevertheless, although tilianin shows remarkable pharmacological importance as a cardioprotection, more studies are required to confirm tilianin’s potential as an agent against CVDs, especially in randomized clinical trials.

## 6. In-Silico Molecular Docking Study of Tilianin

**Figure 7:**
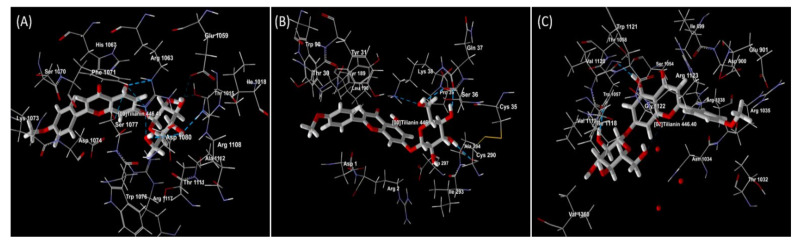
Docked view of Tilianin on (**A**) PDB ID: 5KLB (CavAb voltage-gated calcium channel), (**B**) PDB ID: 6JOD (Angiotensin II type 2 receptor) and (**C**) PDB ID: 7BU6 (Human beta-1 adrenergic receptor).

Moreover in this review, an in silico molecular docking study was conducted by us with CavAb voltage-gated calcium channel, angiotensin II type 2 receptor and human β1 adrenergic receptor to support the cardioprotection potential and mechanism of action of tilianin. Ca^2+^ antagonists are commonly utilised in the treatment of CVDs. 1,4-dihydropyridines are used to treat hypertension and angina pectoris, and they are thought to act as allosteric modulators of voltage-dependent Ca^2+^ channel activation, whereas phenylalkylamines and benzothiazepines are used to treat cardiac arrhythmias, and they are thought to physically block the pore [37]. The renin-angiotensin system (RAS) is blocked, which lowers the risk of cardiovascular events. The cardio-protective benefits of RAS inhibitors are not only dependent on hypertension control. The renin-angiotensin-aldosterone system (RAAS) effector peptide angiotensin II mediates a range of effects on blood pressure and body fluid balance and has been identified as a pathogenic factor at several points along the CVD continuum [38,39]. The sympathetic nervous system and the renin-angiotensin-aldosterone system (RAAS), particularly angiotensin II, suppress NRG-1 expression. RAAS inhibition may enhance cardiomyocyte survival pathways in patients undergoing HER2 inhibitor therapy by lowering angiotensin II levels. Carvedilol, nebivolol, and alprenolol are β-blockers that inhibit the β-1 adrenergic receptor and transactivate β-arrestin. Patients with proteasome inhibitors-related cardiac dysfunction are currently treated with β-blockers and RAAS inhibitors, but patients with light chain amyloidosis often do not tolerate these medications well [40,41]. Hence, all these three targeted protein structures (CavAb voltage-gated calcium channel, angiotensin II type 2 receptor and human β1 adrenergic receptor) were retrieved from the Protein Data Bank and processed for docking analysis using Molegro Virtual Docker 6.0 [42]. The docking results were inspected using the Pose Organizer and the ligand energy inspector tool, and the results were tabulated and the docked view was extracted (Table 2 and Figure 7). It was revealed that the MolDock score with the lowest values had the highest binding affinity to the target proteins. According to the findings, tilianin affinity for CVD protein targets was determined to be 7BU6 > 5KLB > 6JOD. Furthermore, the tilianin molecular docking scores were quite comparable to those of the standard drugs currently used to treat CVDs. This finding supports tilianin’s cardioprotective potential, perhaps advancing this physiologically active molecule to the next stage of drug discovery and development. In silico methodologies are playing an ever-increasing role in drug discovery that are critical in the cost-effective identification of promising drug candidates. However, the in silico result is not reliable to the researchers/scientists since it is a computer-generated simulation result. So, there must be a validation with in vitro and in vivo studies with experimental evidence to prove the data obtained from in silico are trustworthy and reliable. The overall literature evidence of in vitro and in vivo study results of tilianin reported against CVDs are well correlated with the present in silico docking results, which proves the predictability of the in silico model is reliable and can be used in the early stage of drug discovery and development of small molecules for many disorders including CVDs.

## 7. Toxicity Profile of Tilianin

The experimental mice given tilianin at doses up to 1000 mg/kg showed no evidence of toxicity, according to Hernández-Abreu et al. [10]. Experimental animals given doses more than 1000 mg/kg, on the other hand, displayed lethargy for the first 5 h following administration, but then returned to normal. This data indicates that tilianin is not toxic to experimental animals and is completely safe. Furthermore, they found that tilianin has a wide range of pharmacology-toxicology reactions as an effective antihypertensive that is dose-dependent with a low ED_50_ (53.51 mg/kg) compared to the LD_50_ (6624 mg/kg). Using a toxicity estimation software tool with QSAR methodologies consensus method, hierarchical clustering method, and nearest neighbour method, the oral rat LD_50_ mg/kg (predicted value) of tilianin was found to be 1060.12 mg/kg, 755.40 mg/kg, and 2622.95 mg/kg, respectively [43]. All of these findings support that tilianin is toxic free upto the mentioned dose level and should be explored for the development of a drug molecule to treat CVDs. Jiang et al. [16] found that tilianin minimized the impact of OGD/R-induced damage in H9c2 cells in another investigation. Furthermore, there were no differences in cell viability between control and tilianin-treated cells, indicating that the conditions were toxicity-free.

## 8. Pharmacokinetics and Bioavailability of Tilianin

In a study by Yuan et al. [44], tilianin’s pharmacokinetics shows a two compartment model in rats following oral administration of microemulsion. The half-life (t1/2) of tilianin reduced dramatically with increasing dosages (25–50 mg/kg), although the area under the curve [AUC (0–t)], maximum concentration (Cmax) and rate constant (k) were higher than those reported at high doses. The absolute bioavailability of tilianin following oral administration of its microemulsion at 25 and 50 mg/kg, was 3.4% and 3.2%, respectively, while the relative bioavailabilities were 147.2% and 168.2%, respectively. The use of microemulsion can markedly increase the bioavailability of tilianin [44]. Acacetin-7-glucuronide and sulfate are the primary metabolites of tilianin detected in mouse plasma where the latter is found in higher concentrations in Bcrp1 type FVB mice (−/−) when compared to wild-type FVB mice. In the near future, more pharmacokinetics and bioavailability studies on tilianin are needed to obtain a greater understanding of its absorption, distribution, metabolism, and excretion (ADME) abilities. Furthermore, improved bioavailability of tilianin is likely to propel this promising natural molecule to the forefront of therapeutic medicines for the treatment of human disease, particularly CVDs.

## 9. Challenges and Opportunities of Tilianin to Be Developed as a Drug Molecule for the Treatment CVDs

Plant-based drug discovery initiatives continue to be key sources of new drug indicators, despite a number of hurdles such as obtaining and verifying plant materials, implementing high-operational evaluation bioassays and the yield of bioactive lead molecules. Beyond scientific discoveries and innovations, the barrier to developing new cardiovascular medicines is tremendous. Despite the fact that CVD is becoming more prevalent in developing countries, there is still time to prevent the pandemic from reaching its maximum potential. In this context, there are challenges and opportunities for tilianin to become a drug molecule for CVD treatment. Tilianin looks to be a promising drug-like molecule with the potential to be a good therapeutic agent for a variety of disorders, including CVDs, according to data acquired from the DruLiTo software [45] (Table 3). One of the challenges is the commercial availability of tilianin. As stated by Wang et al. [46] some tilianin flavonoids namely acacetin-7-glucuronide and sulfate are extremely limited. Furthermore, although tilianin has a protective mechanism on atherosclerosis [29], it remains unclear in some studies. Nevertheless, to date, there is no definitive study that stated the fact that it can be used as an anti-atherosclerotic agent and more studies are recommended [30] to point its role in this direction. In addition, there is yet insufficient direct confirmation for tilianin’s potential to reverse endothelial cell dysfunction, especially in view of the fact that the pathology and risk factors that lead to the onset of CVDs are broad. Based on the literature reviewed, tilianin is confirmed to have a significant cardioprotective mechanism [17,21,22,23,24,25] and anti-hypertension [34]. Nevertheless, although tilianin has various therapeutic potentials against CVDs, the majority of studies remain in the preclinical stage, where the availability of randomised controlled trials can help confirm the findings.

**Figure 8:**
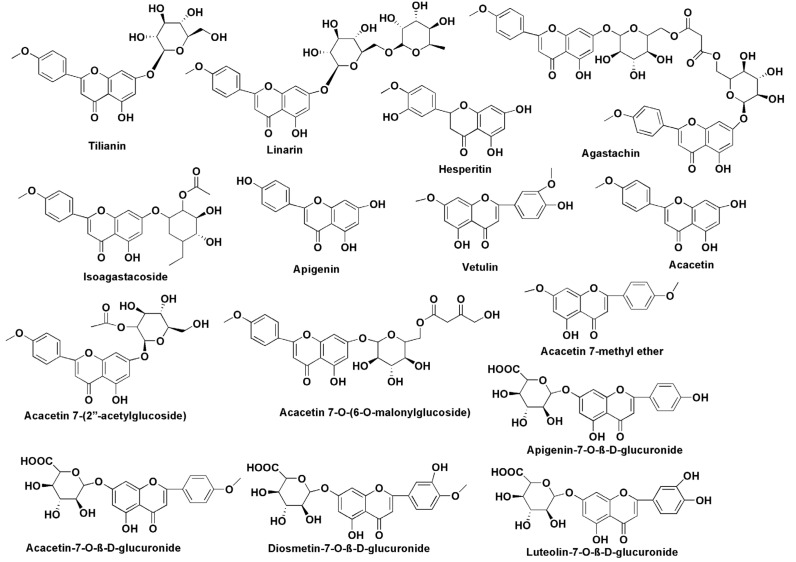
Chemical structure of tilianin derived/related compounds.

**Figure 9:**
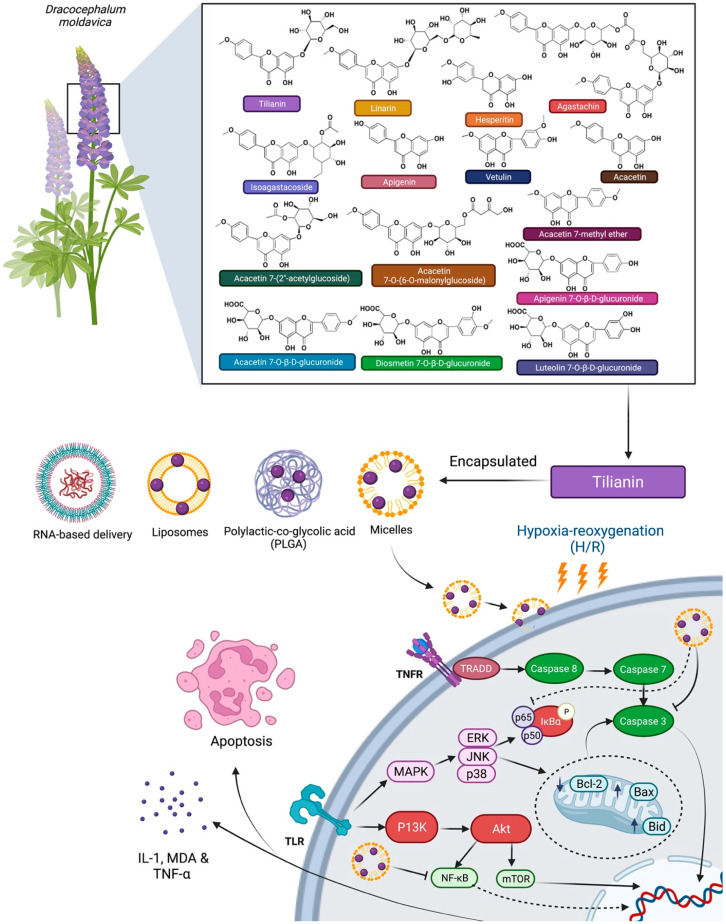
Possible drug delivery methods of tilianin enclosed within nanocarriers, i.e., micelles, to increase its anti-apoptotic effect. Abbreviations: TLR, Toll-like receptor; TNFR, Tumor necrosis factor receptor; MAPK, Mitogen-activated protein kinase; P13K, Phosphatidylinositol-3-Kinase; ERK, Extracellular signal-regulated kinase; JNK, c-Jun N-terminal kinases; IκBα, Nuclear factor of kappa light polypeptide gene enhancer in B-cells inhibitor alpha; Akt, Ak strain transforming; NF-kB, Nuclear factor kappa-light-chain-enhancer of activated B cells; mTOR, Mammalian target of rapamycin; Bcl-2, B-cell lymphoma 2; Bax, Bcl-2 Associated X-protein; Bid, BH3 interacting-domain death agonist; IL-1, Interleukin-1; TNF-α, Tumour necrosis factor alpha; MDA, Melanoma differentiation associated.

In addition, structural changes within tilianin can improve its physicochemical, bioavailability, and pharmacokinetic properties. Molecules related to tilianin have changes in the 7th position of the flavonoid ring where the glycoside ring is attached. Major non-volatile metabolites related to tilianin are hesperidin, apigenin, acacetin, linarin, isoagastachoside, agastachin, acacetin 7-(2″-acetylglucoside), acacetin 7-O-(6-O-malonylglucoside), acacetin 7-methyl ether, vetulin, luteolin-7-O-*β*-D-glucuronide, apigenin-7-O-*β*-D-glucuronide, diosmetin-7-O-*β*-D-glucuronide and acacetin-7-O-*β*-D-glucuronide (Figure 8) [47,48,49,50]. Hesperidin is one of the tilianin-related molecules that has been extensively researched against CVDs [51]. Apart from that, apigenin and acacetin have been associated with reduction in CVD risk [30,52]. In an attempt to increase its bioavailability, tilianin has been encapsulated in a hydrophobic shell to produce micro micelles [46]. Tilianin loaded micelles (70 nm) are effective hydrogen peroxide scavengers that also inhibit caspase-3 activity, thereby protecting cells from H/R-induced cytotoxicity. In a hypoxia-reoxygenation (H/R) model, tilianin loaded micelles lowered MDA, IL-1 and TNF-α, inhibited apoptosis, TLR4 and NF-κB p65 protein expression. By improving drug targeting, pharmacokinetics, efficacy and cellular uptake, nanomedicine has the ability to bridge the gap between pharmaceutical restrictions and natural phytochemical therapeutic potentials [53,54,55,56,57,58]. Nanoparticles in drug delivery such as polylactic-co-glycolic acid (PLGA), polyethylene glycol (PEG), liposomal delivery methods, and RNA-based delivery have various advantages, including: (1) enhanced adsorption; (2) selective targeting; (3) simple encapsulation (4) an increase in bioavailability; (5) decrease in adverse effects and (6) enhanced stability for CVDs [59,60,61]. These new delivery systems provide a number of approaches for achieving tissue selectivity and reducing system exposure, which are both necessary for deploying new pharmacological compounds derived from tilianin for improved CVD treatment (Figure 9).

## 10. Conclusions and Future Perspectives

**Figure 10:**
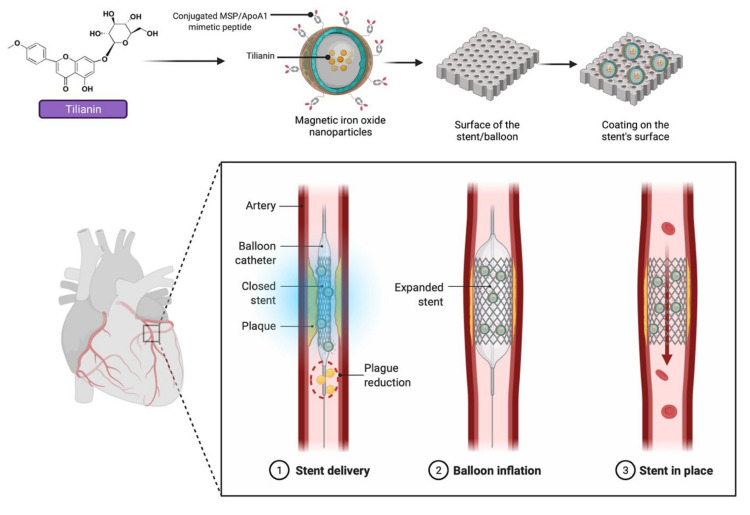
Tilianin-loaded magnetic iron oxide nanoparticles augmented with surface decorated MSP/ApoA1, deposited onto the surface of the stent/balloon for in situ targeting approach for effective plague reduction activities in the future.

Nature has provided many drug molecules that are currently on the market. Furthermore, natural products from terrestrial sources (plants and fungus), microorganisms, and marine species, have been employed to provide a lead molecule for a new drug design, development, and therapy. Overall, the current review reported that tilianin is a potential natural lead molecule against CVDs including coronary heart disease, angina pectoris, hypertension and myocardial ischemia. Tilianin’s protective effects are attributed to several mechanisms notably free radical scavenging, inflammation control, mitochondrial function regulation and related signalling pathways. To understand the role of tilianin in the treatment of peripheral artery disease, cardiomyopathies, coronary artery disease, ischemic stroke, dyslipidemias, aortic aneurysm, diabetic cardiovascular complications, atherosclerosis, cardiac hypertrophy, and heart failure, future research should focus on the exploration and integration of innovative, organically generated drug prospects in the health and therapeutic sectors. Altogether, the findings of this review indicate that tilianin appears to be a promising molecule for new drug development in the prevention and treatment of CVDs. However, further preclinical research on tilianin and its derivatives is needed to validate its potential benefit and mechanism in the prevention of CVDs. Pharmacokinetic and bioavailability studies are also required before this molecule may be used in drug development. In terms of drug delivery, magnetic nanoparticles have recently sparked interest, particularly for those utilizing drug-eluting stent (DES) technologies, as they potentially can cause a paradigm shift with major therapeutic improvements. Earlier research has indicated that these strategies minimise the risk of reobstruction after stenting, a condition known as in-stent restenosis. Nevertheless, current DESs have some limitations such as the reduced bioavailability and the inability to alter the drug’s dosage and pharmacokinetics, especially imposed by the variability in the disease condition of the treated artery. A proposed strategy for overcoming the addressed limitations is to use magnetic iron oxide nanoparticles conjugated with MSP/ApoA1, a synthetic gene derived from a component of high-density lipoprotein (HDL), which can aid in preventing inflammation, oxidative stress and cholesterol efflux to reduce arterial fat formation that can lead to blockage. As discussed in the review, incorporation of tilianin may assist in minimising the growth of ox-LDL-induced foam cells and prevent further acceleration of the signalling cascade involved in atherosclerosis formation. The nanoparticles chosen can increase the utility of tilianin’s entrapment and distribution. Due to their physical features, they may be deposited onto the surface of a stent/balloon and dissociated for release for optimum localised drug administration to the afflicted area (Figure 10). We anticipate that the strategy can be a novel approach to drug delivery technology. Altogether, we believe that tilianin is a potential natural lead molecule for a new drug design, development, and therapy for CVDs, based on the scientific evidence presented in this review.

## Figures and Tables

**Table 1:** A summary of in vivo studies investigated the efficacy of tilianin against CVDs.

Animal, Sex	Model	Dose of Tilianin Dose (mg/kg/Day)	Route of Administration	Duration of Treatment	Mechanism of Action	Therapeutic Effects	References
SD rats, male	Isolated heart ischemia/reperfusion	--	--	--	- By inhibiting CaMKII-mediated mitochondrial apoptosis - By inhibiting JNK/NF-*κ*B inflammation	Cardioprotection	Jiang et al. [21]
SD rats, male	Myocardial ischemia reperfusion injury	5	Intragastic	7 Days	- By improving mitochondrial energy metabolism - By reducing oxidative stress via AMPK/SIRT1/PGC-1α signaling pathway	Cardioprotection	Tian et al. [22]
SD rats, male	Myocardial ischemia reperfusion injury	2.5, 5, 10	p.o.	14 Days	- By activating the PI3K/Akt signalling pathway - By inhibiting myocardial apoptosis	Cardioprotection	Zeng et al. [17]
SD rats, male	Myocardial ischemia reperfusion injury	1.25, 2.5, 5	p.o.	7 Days	- By alleviating apoptosis of cardiomyocytes - By protecting myocardium through protection of mitochondria and repression of mitochondrial apoptotic pathways	Cardioprotection	Wang et al. [23]
SD rats, male	Myocardial ischemia reperfusion injury	1.5, 2.5, 5	p.o.	7 Days	- By relieving calcium overload - By correcting energy metabolism - By improving endothelial function - By inhibiting cell apoptosis	Cardioprotection	Guo et al. [24]
SD rats, male	Myocardial ischemia reperfusion injury	1.25, 2.5, 5	p.o.	7 days	-By raising the levels of ATP of the myocardium - By protecting micro-structures and functions of mitochondria	Cardioprotection	Yuan et al. [25]
*Ldlr*−/− mice, male	High-cholesteroldiet	high-cholesterol diet supplemented with 0.05% (*w/w* diet) of tilianin	p.o.	7 weeks	- By inhibiting NF-κB-dependent pro-inflammatory cytokines (TNF-α and IL-1β) - By inhibiting IκB kinase activity	Atheroprotection	Nam et al. [28]
SHR rats, Male	Spontaneously hypertensive rats	50	p.o.	Single dose	- By endothelium-dependent manner, probably due to NO release - By endothelium-independent pathway by opening up K^+^ channels	Anti-hypertensive	Hernández-Abreu et al. [34]

The main body text covers all of the abbreviations.

**Table 2:** Docked study results of tilianin and standard drugs with the CVD target proteins.

S. No.	Protein Data Bank ID	Name of the Protein	Ligand	MolDock Score	Rerank Score	HBond
1.	5KLB	CavAb voltage-gated calcium channel	Tilianin	−106.31	−96.8228	−14.7418
			Amlodipine	−129.418	−40.768	−1.49788
			Verapamil	−86.916	−42.9639	0
2.	6JOD	Angiotensin II type 2 receptor	Tilianin	−97.5636	−87.5858	−8.44394
			Azilsartan	−124.389	−77.9133	−9.92896
			Losartan	−116.448	−51.6799	−8.43829
3.	7BU6	Human β1 adrenergic receptor	Tilianin	−115.036	−95.7438	−5.83387
			Nebivolol	−100.61	−80.3808	−5.56831
			Atenolol	−84.7246	−65.7884	−6.11871

**Table 3:** Physicochemical and drug-likeness properties of tilianin.

Property/Rule	Result
Molecular formula	C_22_H_22_O_10_
Molecular weight	446.12
Hydrogen bond donors	5
Hydrogen bond acceptors	10
Rotatable bonds	5
*Log P* (Partition coefficient, Predicted value)	0.153
Molar refractivity	117.89 cm^3^
Topological polar surface area	155.14 Å^2^
Lipinski’s Rule of Five	Passed
Unweighted QED	Passed
Weighted QED	Passed

QED, Quantitative estimate of drug-likeness.

## Data Availability

The data presented in this study are available on request from the corresponding author.
